# A Critical and Historical Discourse Regarding Gentili Da Fuligno, a Celebrated Physician of the 14th Century

**Published:** 1846-01

**Authors:** 


ART. VII.—*Sopra Gentile da Fuligno, Medico Illustre del Secolo XIV.* Discorso Storico-critico del Dottor GIUSEPPE GIROLAMI, Medico in Civita-Vecchia.—*Napoli,* 1844.

*A Critical and Historical Discourse regarding Gentili da Fuilgno, a celebrated Physician of the* 14*th century.* By Dr. G. GIROLAMI, &c. 8vo, pp. 60.

ITALY was full of popular cities, and abounded in all the knowledge and arts which emanate from popular cities at a period when the rest of Europe was but half civilized. Amongst those learned men, who constituted the avant-couriers of modern philosophy, Gentili da Fuligno was conspicuous. He was the son of a celebrated physician and writer of Fuligno, (from which city the family surname was derived), who was born there in 1230, and died at Bologna in 1310. Gentili was educated at Bologna, the pupil of the celebrated Taddeo di Fiorenza, who was professor there from 1260 to 1295, and an enthusiastic student of the Hippocratic books. At Bologna, Gentili became distinguished for his learning, and was chosen to fill the chair of medicine at Bologna and Perugia. Both these cities conferred upon him the right of citizenship, and Perugia, in addition, presented him with a house situate near the church of the Augustines. He was also professor at Padua during the years 1337 to 1345. Ubertino da Carrara, lord of Padua, having fallen sick, sent for him to Padua, and that he might have Gentili near him, presented him with the professorship. It is supposed Gentili was also physician to that same Pope John 22d, who had Petrarch for his secretary, and by him was loaded with wealth and honour.

In the year 1340, the black death devastated Italy. The contagion was brought by a Genoese vessel to Genoa, from whence it spread to the adjoining towns, arriving at Perugia in April. It there broke out with all the symptoms of the Levant plague. It is not certain whether Gentili went expressly to that city to assist its inhabitants in grateful return for the honour and property they had bestowed upon him, or whether he was already there occupied with the duties of his professorship. Dr. Girolami inclines to the latter opinion. Be this as it may, Gentili devoted himself most assiduously to the duties of his calling. He immediately wrote his "Consilium de Peste," for the instruction of the citizens; he recommended and carried out such measures of public hygiene as he thought necessary; and at last, wearied with incessant attendance on the sick, was himself attacked with the plague, and died in six days. The manuscript copy of his "Consilium de Peste," found in the Malatesta Library at Cesena, has the following annotation by a pupil at its close:

"Et postea Gentilis infirmatus est ex nimia requisitione infirmorum, et hoc fuit 12 die Junii, et vixit sex diebus, et mortuus est, cujus anima requiescat in pace. Hoc fuit MCCCXLVIII. Et ego Franciscus de Fulgineo interfui ægritudini ejus, et numquam dimisi eum usque ad mortem, et sepultus fuit Foligini in loco Eremitarum." (p. 10.)

There is here a charming touch of human kindness. In the midst of despair and death, amongst the arrows that flew at noonday, and the pestilence that walked in darkness, the master of the medical art, led on the van of his small and feeble array. He sinks, overpowered; but his pupil, regardless of the danger, is with him to the end, and on the dusty MS. of his master records the fact, "I, Francis of Fuligo, attended him in his sickness, and left him not until he breathed his last." And when the tumult of pestilential destruction was sunk into the stillness of death, the same faithful hand removed his remains to his native soil.

Gentili wrote several works, &c. A comment on the first three canons of Avicenna; Consilium de Peste; De Febribus; Consilia Peregregia, or a Collection of Cases; a tract on Hernia; Recepta; De Balneis; Quæstiones subtilissimæ in Artem parvam Galeni; De Proportionibus Medicinarum, &c.; Expositio cum Commento Mag. Egidii Monachi Benedictini, lib. i.; (an example of a monk-physician;) Judiciorum de Urinis; De Lepra Tractatus; De Balneis Tractatus. The scientific bibliographer will find the titles and editions of these books with other information regarding them in Dr. Girolami's pamphlet. There is also an account of the social, political, and literary condition of Italy, at the time in which Gentili flourished,—a topic always pleasing to the Italian,—for the contemplation of the past glory of his country affords him hope and solace when he views its present abasement.

